# A practical method for storage, preservation and transportation of anuran urine samples using filter paper for hormone analysis

**DOI:** 10.1016/j.mex.2021.101578

**Published:** 2021-11-12

**Authors:** Vinod Kumar, Shudhanta Sood, Karthikeyan Vasudevan, Govindhaswamy Umapathy

**Affiliations:** Laboratory for the Conservation of Endangered Species (LaCONES), CSIR-Centre for Cellular and Molecular Biology (CCMB), Hyderabad, Telangana 500048, India

**Keywords:** Progesterone, Testosterone, Non-invasive, enzyme immunoassays, HPLC, Field method, Asian common toad

## Abstract

Anurans (frogs and toads) expelled urine when handled and it could provide insights into their physiological status. However, storage, preservation and transportation are often challenging. The study aimed to standardize and validate a field method for short-term storage and preserve of anuran urine samples using Whatman filter papers. To examine the efficacy of storage conditions and type of papers, urinary based enzyme immunoassays were used to measure progesterone and testosterone hormone metabolites. High-Performance Liquid Chromatography was performed and revealed immunoreactive progesterone and testosterone metabolites in the urine samples. Urinary hormone metabolites concentration stored in filter paper at room temperature and control samples stored in -20°C for the same period were similar. Whatman grade 50 was found to be more suitable for storage of hormones than grade 3 paper for the experiments performed.

• A cheap and simple storage method for storage of anuran urine in field conditions using filter papers.
• Anuran urine could be preserved and transported under ambient conditions without significant changes and loss of hormones.
• This method would facilitate the endocrine monitoring of anurans in remote areas where limited logistics are available.

## Graphical abstract

**Figure:**
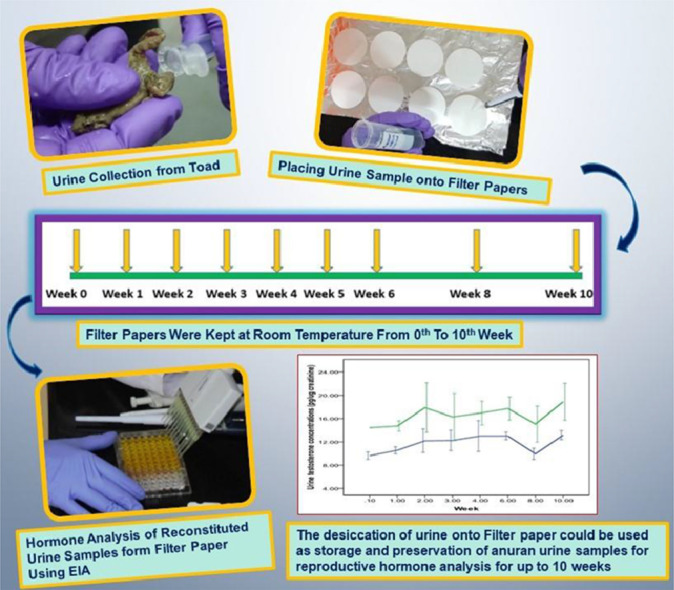

Specifications table

**Table:** 

Subject Area:	Agricultural and Biological Sciences
More specific subject area:	Conservation physiology of anurans
Method name:	A field storage method of anuran urine on filter paper for hormone analysis
Name and reference of the original method:	C.D. Knott, Radioimmunoassay of estrone conjugates from urine dried on filter paper. Am J Primatol, 67(2005) 121-135.
Resource availability:	Yes, for hormones (https://www.steraloids.com) filter paper (https://www.cytivalifesciences.com).

## Introduction

Among 8100 described species of amphibians recorded in the world 41% are threatened with extinction, as they are categorized as critically endangered, endangered or vulnerable (IUCN 2020) [1]. Amphibians, the most threatened vertebrates, are severely affected because of habitat loss, pollution (including pesticides), over-harvesting, infectious diseases (such as *Chytridiomycosis*), invasive species, and climate change (including an increase in UV-B radiations) [2], [3], [4]. These factors have synergistically acted and pushed many species to the brink of extinction. The IUCN Red List (2008) showed that about 122 species of amphibians have not been reported since the 1980s, and nearly one-third of species are threatened [5]. Amphibians play many vital roles in an ecosystem and are critical links in food webs. In both tropical and temperate environments, amphibians may exceed all the other terrestrial vertebrate classes (reptiles, birds, and mammals) in terms of the number of individuals and the proportion of total biomass available [6]. India supports rich amphibian fauna consisting of 384 species, and nearly 80% of them are endemic to the country [7]. India also ranks among the top countries with the highest number of threatened amphibians [2]. In response to the amphibian biodiversity crisis, several important interventionist conservation actions have been recommended, including the establishment of *ex-situ* captive breeding and reintroduction programs [8]. Incorporating reproductive technologies for amphibian captive breeding and reintroduction programs are essential to improve the reproductive health of individuals [9].

The endocrine control of ovulation and sperm release in amphibians is poorly understood. However, this knowledge is fundamental for careful application and development of hormone treatments to control reproduction in captive amphibians [9]. To perform endocrine studies in a captive breeding set up collection of blood or tissues from anurans (frog and toad) is not feasible. Prolonged restraining of anurans for such procedures could lead to an increase in their stress response [10,11]. Steroid hormones in urine represent their cumulative excretion over several hours [12]. They are more resistant to fluctuations observed previously in serum hormone concentrations [13]. However, urinary steroid hormones are unstable at room temperature and require immediate freezing for their preservation. An easy method for storage, preservation and transporting urine samples in the dry state is essential for anuran endocrine studies. In this study, we attempt to standardize a method for short-term storage and preservation of anuran urine using filter papers. For this purpose, we used two different grades filter papers (Whatman grade 3 and 50, which are known for high retention and absorbent characteristics, respectively) to standardize storing and preserving of anuran urine samples.

### Sample collection

Urine samples were collected from free-living the Asian common toad (*Duttaphrynus melanostictus*) at Laboratory for the Conservation of Endangered Species, Centre for Cellular and Molecular Biology campus, Hyderabad, India, between January and March 2016. We collected urine sample non-invasively by gently holding on to our palms. The anurans urinate as soon as they captured [9]. We collected urine samples directly into labelled glass beakers and transferred them into Eppendorf tubes. The animal handlers have used sterile gloves while collecting samples, and after every collection, the gloves were disposed off to avoid any contamination between samples.

### Whatman filter paper

We studied Whatman paper grade 3 (CAT No. 1003-055) and grade 50 (CAT No. 1450-055) filter papers from GE Health-care Life Sciences for the storage experiments. The characteristics of the papers are given in Table 1.

**Table 1:** Characteristics of Whatman filter paper grade 3 and 50 used in experiments.

Characteristics	Whatman paper grade 03	Whatman paper grade 50
Thickness	390 μm	115 μm
Diameter	55 mm	55 mm
Pore size	6 μm	2.7 μm
Material	qualitative cellulose filter paper	cotton linters filter paper
Wet strength	Low	High
Retention	Low	High

### Storage and preservation of urine on filter paper

1. Toad urine samples were pooled and mixed thoroughly. About 40 ml of urine sample was collected from 30 toads. For the control experiment, a set of samples were immediately preserved at -20 °C until further analysis.
2. To examine the storage and retention of urine samples, two types of Whatman grade 3 and 50 filter papers were used for up to 10 weeks. Each week had a subset of 6 samples for both the grade papers. These papers were labelled with unique IDs.
3. Each labelled Whatman filter paper was placed on a non-absorbent surface (aluminium foil) and 200 μl of freshly pooled urine sample was then applied using a micro-pipette. A total of 108 subset samples were prepared for the experiment, which includes 0 – 10 weeks.
4. The samples were then allowed to dry at room temperature for two days by placing the aluminium foil, along with the filter papers, in air-tight containers filled with silica gel beads.
5. After drying, each filter paper was placed in a separate plastic zip-lock (which had holes in it) and marked for sample identity, date and weekly number.
6. These zip locks were then placed in a container filled with silica gel beads for short-term storage.
7. The storage containers were kept at room temperature and away from light and heat.
8. Every week, the respective samples were removed from their storage container and reconstituted with 5 ml of 100% methanol in a screw-topped glass tube to allow for the elution of hormonal metabolites from filter paper and kept at 4°C for 2 h.
9. The Whatman papers were removed following complete squeezing out of methanol using sterile forceps that were cleaned with 100% methanol between each use.
10. The glass tubes were then placed under the stream of nitrogen gas to allow the evaporation of methanol.
11. After evaporation of methanol, 1000 μl of EIA buffer (equivalent to 1:5 dilutions of the original urine sample) was added to each test tube and vortexed thoroughly for 2–3 minutes.
12. The urinary extract was then stored at -20°C until further analysis.
13. This protocol was followed for all samples up to 10 weeks and respective replicates.

### Urinary steroid hormones analysis using enzyme immunoassays

1. Urinary progesterone metabolites were measured using previously developed progesterone EIA [14,15]. In this assay, we used a monoclonal progesterone antibody (CL425, provided by Dr. Coralie Munro, University of California, USA.), progesterone conjugated HRP (horseradish peroxidase) and progesterone standards (200 pg/well to 0.39 pg/well). The antibody cross-reacts with progesterone (100%) and with other metabolites as determined by Graham et al. 2001 [16].
2. Similarly, previously developed testosterone EIA was used to measure urinary testosterone. We used polyclonal testosterone antibody (R156/7, Provided by Dr. Coralie Munro, University of California, USA), testosterone conjugated HRP and testosterone standards (600 pg/well to 1.17 pg/well) for this assay. The antibody cross-reacts with dihydrotestosterone 57.4%, <0.3% with androstenedione and <0.1% with androsterone, dihydroepiandrosterone, β-estradiol and progesterone [14,15].
3. The 96-well Nunc MaxiSorp microtiter (Thermo Fischer Scientific, UK) high binding plate was coated with 50 μl of diluted antibody with coating buffer (0.05M sodium bicarbonate buffer at pH 9.6) in each well and kept at 4°C overnight in an airtight, humid container.
4. Following incubation, the plate was washed with an automatic plate washer (Elx50; BioTek, USA) for four times using wash buffer (0.15M NaCl, 0.05% Tween20) and dried on a paper towel. Subsequently, 50 μl of urine samples or respective standards diluted in EIA buffer (0.1 M PBS containing 0.1% BSA at pH 7.0) were added followed by 50 μl of conjugated HRP in each well and incubated at room temperature for 2 hrs.
5. The plate was rewashed after incubation and 50 μl of Tetramethylbenzidine/H2O2 (TMB, Genei, India) was added to all well and incubated in the dark until the average optical density reaches 0.8-1.0 in the zero well.
6. The reaction was stopped by adding 50 μl of 1N hydrochloric acid (HCl) and absorbance was measured at 450 nm Multiskan Spectrophotometer (Thermo Multiskan Spectrum Plate Reader, version 2.4.2; Thermo Scientific, Finland)
7. Based on the absorbance values obtained from the known concentrations, a standard curve was plotted. Based on Beer-Lambert's law, this standard curve was used to obtain the concentration of the unknown samples.

### Creatinine assay

Creatinine is excreted at a constant rate in urine following protein metabolism. Therefore, it is used as a concentration index against hormonal metabolites measured in urine. It was quantified by using the modified Jaffe reaction [17]. Creatinine concentrations show the time over which hormones are metabolized and excreted into the urine, regardless of the volume of urine. Urine hormone concentrations are expressed in relation to creatinine concentrations [10].

The procedure for the creatinine assay was as follows:

1. Standards of 500, 250, 125, 62.5 and 31.25 ng/well were prepared from a working stock of 10 μg/mL using milli-Q water for dilution. Urine samples were also diluted 1:4 using milli-Q water.
2. 50 μl of standards and diluted samples were loaded into a 96-well plate.
3. Alkaline picric reagent was prepared immediately before use was then added. It was prepared by combing 4 ml of 0.75 N NaOH, 4 ml of 0.13% picric acid and 4 ml of milli-Q water.
4. The plate was briefly tapped and incubated for 30 minutes at room temperature.
5. Plates were then read at 490 nm in the Multiskan IT Spectrophotometer (Thermo Multiskan Spectrum Plate Reader, version 2.4.2; Thermo Scientific, Finland) and urine creatinine concentrations were estimated.

### High-performance liquid chromatography

High-performance liquid chromatography (HPLC) was used for the separation and identification of immunoreactive progesterone and testosterone metabolites in urine samples using Shimadzu CTO-10AS (Shimadzu Corporation, Japan). Pooled urine samples were purified using the Sep-Pak C18 cartridges (Waters, USA) before injecting into HPLC. The pooled urine sample was passed through the C18 cartridges and eluted with 5 ml of absolute methanol, as described previously [18]. A steroid specific reverse phase C18 column (Waters column, Symmetry C18, 4.6 × 20 mm, 3.5 mm, Intelligent Speed [IS] column) was used for the separation and identification of urinary steroid metabolites. The respective standards were used as reference controls (Steraloids, USA) and urinary metabolites eluted using a gradient of 20 to 64% acetonitrile: water at a flow rate of 2 mL/min: run time 8 minutes. Every 15 s, fractions (250 ul) were collected manually, dried and reconstituted in EIA buffer to determine the immunoreactivity of urinary steroid metabolites with the corresponding antibody used in the assay.

### Data analysis

Recovery rate of urine hormone concentration from the paper was calculated percentage (Recovery rate in percentage = Original hormone concentration - measured hormone concentration from the paper) / original hormone concentration X 100). Data on hormone values were presented as mean ± SD. We also calculated relative standard deviation (Co-efficient of variation, CV) for inter and intra week variations. Mann–Whitney U test (M–W test) was used for testing differences in hormone concentrations between weeks. Kruskal Wallis test (K-W test) was used for testing differences across weeks. The urine hormone concentration of each sample was calculated from the standard curve and expressed as pg/μg of creatinine. All statistical analyses were performed using SPSS 17.1 ver.

## Result and discussion

### Validation of enzyme immunoassays

1. Testosterone and progesterone EIAs were validated by using parallel displacement curves between pooled urine and respective standards (Fig. 1).

**Fig. 1:**
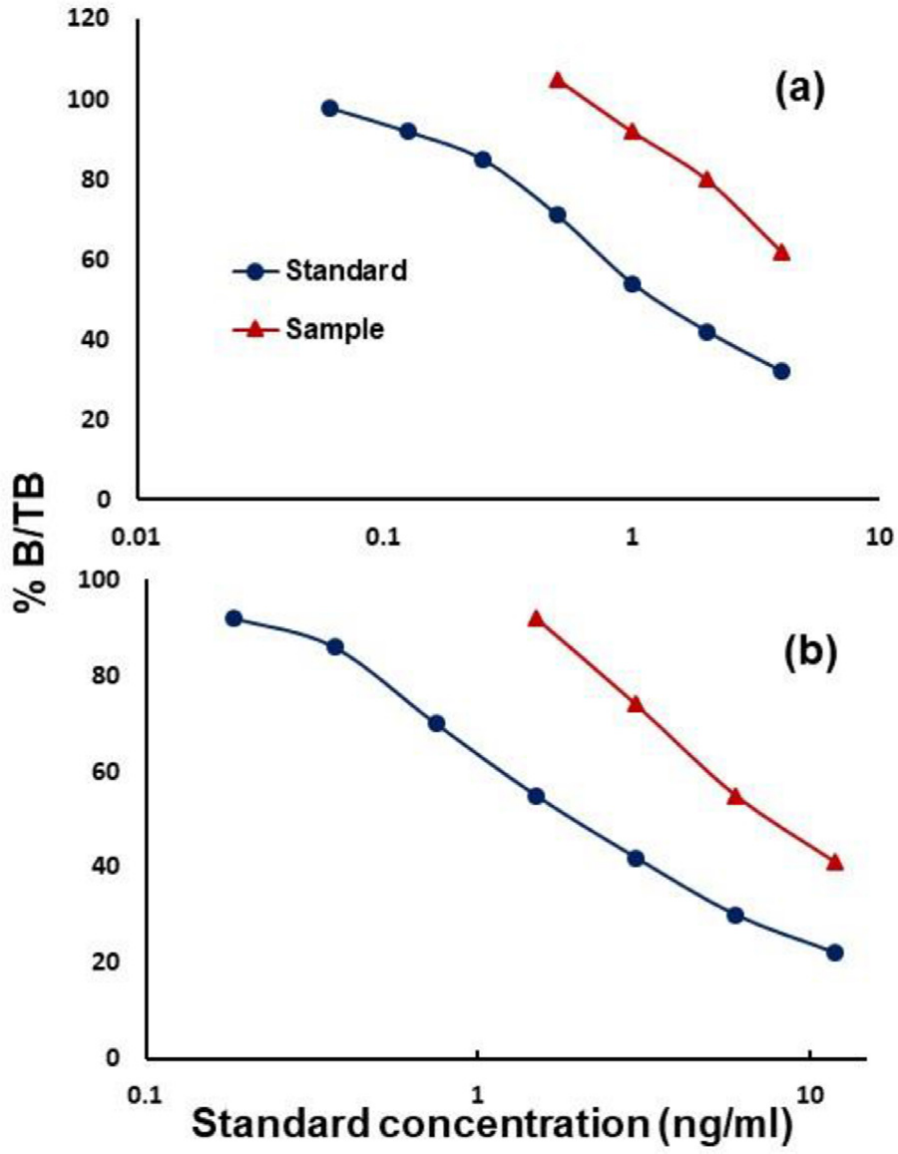
Parallel displacement curves between pooled serially diluted anuran urine sample (triangles) and standard (circles) of (a) Progesterone (b) Testosterone
2. Parallelism curves were made to determine optimum urine sample dilution at 50% binding for testosterone and progesterone. They also showed the immunological similarity between urine steroids and respective standards.
3. The sensitivity of antibodies was calculated at 90% binding and found to be 0.39 pg/well for progesterone and 1.17 pg/well for testosterone, respectively.
4. The inter and intra-assay coefficient of variation (CV) for progesterone and testosterone were 12.26% and 8.93%, and 10.34% and 7.34, respectively.
5. HPLC confirmed the presence of progesterone and testosterone in the pooled urine samples of anuran and eluted fractions confirmed the immunoreactivity with the corresponding antibody used in the assay (Fig. 2).

**Fig. 2:**
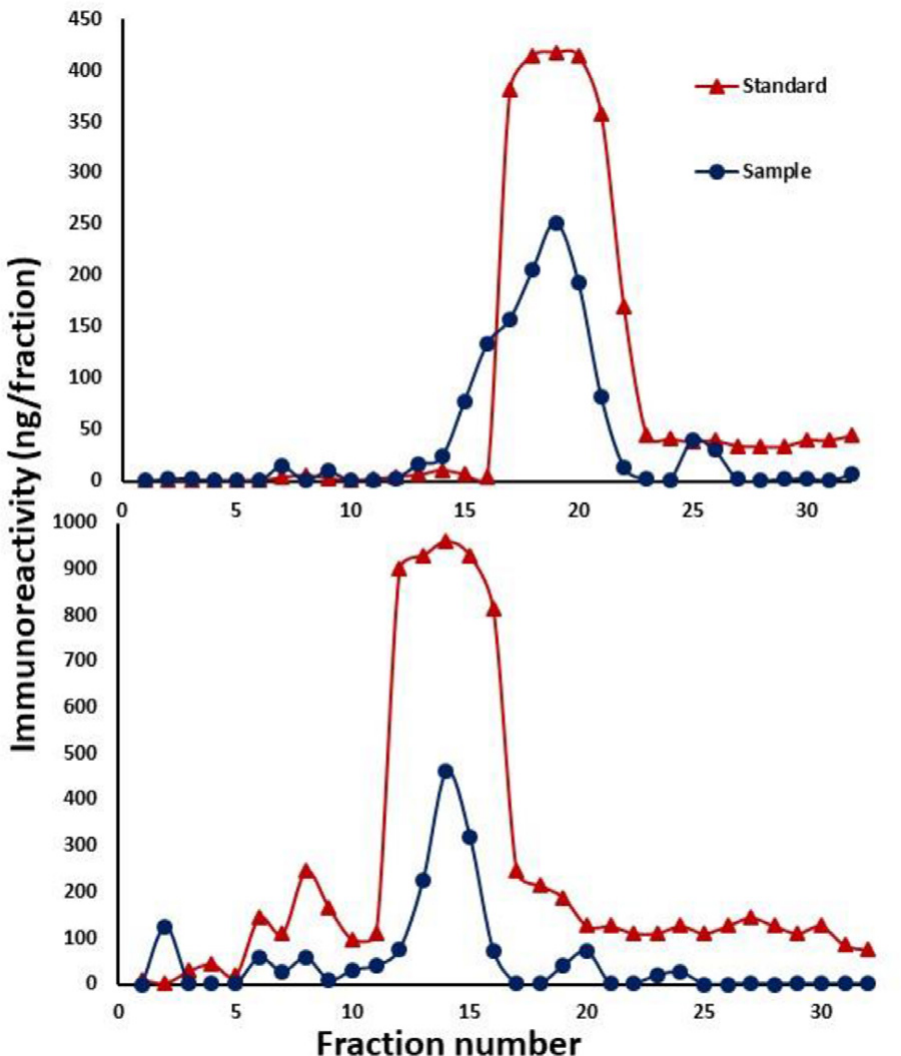
HPLC separation of immunoreactive (a) Progesterone and (b) Testosterone in pooled urine samples (circle) and standards (triangle).

### Testosterone metabolites on whatman 3 and 50

**Fig. 3:**
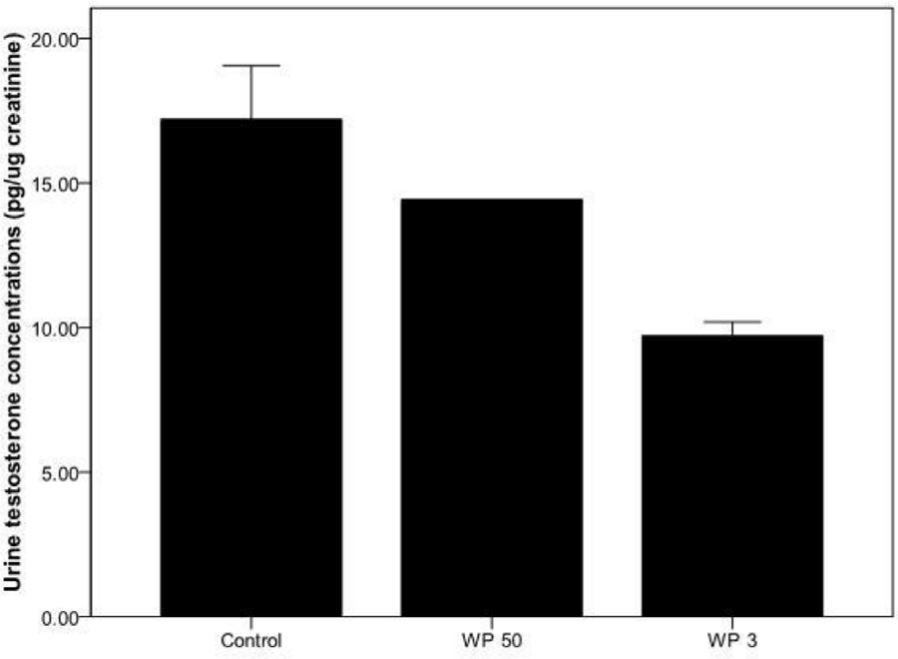
Urinary testosterone metabolites concentration in control (frozen urine) and 0th day Whatman 50 and 3 filter papers. Bars represent mean and standard deviation.

To examine the loss of urine hormones in the paper following the extraction procedure, we compared the stored samples with control samples on the same day. Urine testosterone concentrations varied significantly among the control (stored -20°C) and samples stored in WP 3 and WP 50 (K W test X-12.97, *P* = 0.002; Fig. 3). The sample stored directly in -20°C had significantly higher urine testosterone concentrations than in samples stored in WP 3 and WP 50 paper (MW U test, *p* = 0.001; *p* = 0.01, respectively, Fig. 3). About 56.45% and 85.0% of urine testosterone (compared to control) were recovered from WP3 and WP 50 papers, respectively.

**Fig. 4:**
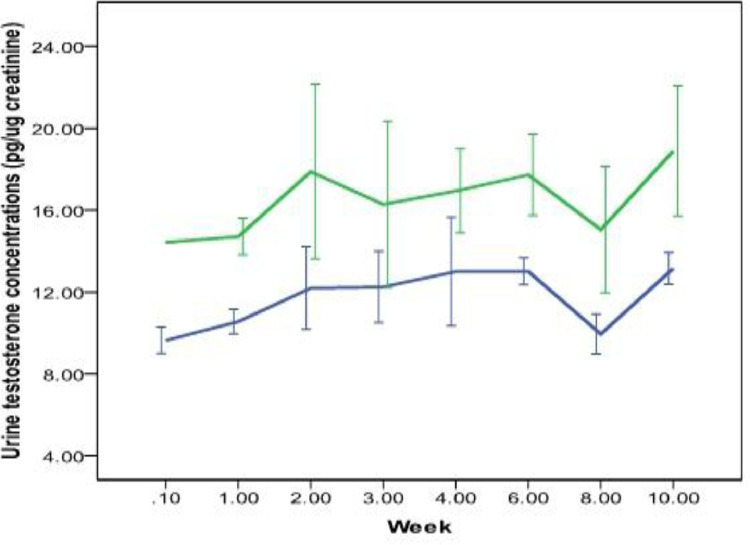
Urinary testosterone metabolites concentration from 0th week to 10th week stored on Whatman 50 (green line) and 3 filter papers (blue line). Bars represent mean and standard deviation.

Between two types of storage papers, the WP 50 had higher urine testosterone concentrations across all the weeks compared to WP 3 (MW U test *p* = 0.02; Fig. 4). Interestingly, significant increases in urine testosterone were also observed in both types of papers following 10 weeks of preservation (*T* = 14.27, *p* = 0.001, *T* = 4.27, *p* = 0.02 for WP 3 and WP 50 paper respectively, Fig 4.). The intra-week co-efficient variations ranged from 3.1 to 10.6 while the inter week variation was 14.1 for WP 50. In the case of WP 3 the intra-week co-efficient ranged from 3.4 to 15.1, while the inter week variation was 17.4.

### Progesterone metabolites on whatman 3 and 50

**Fig. 5:**
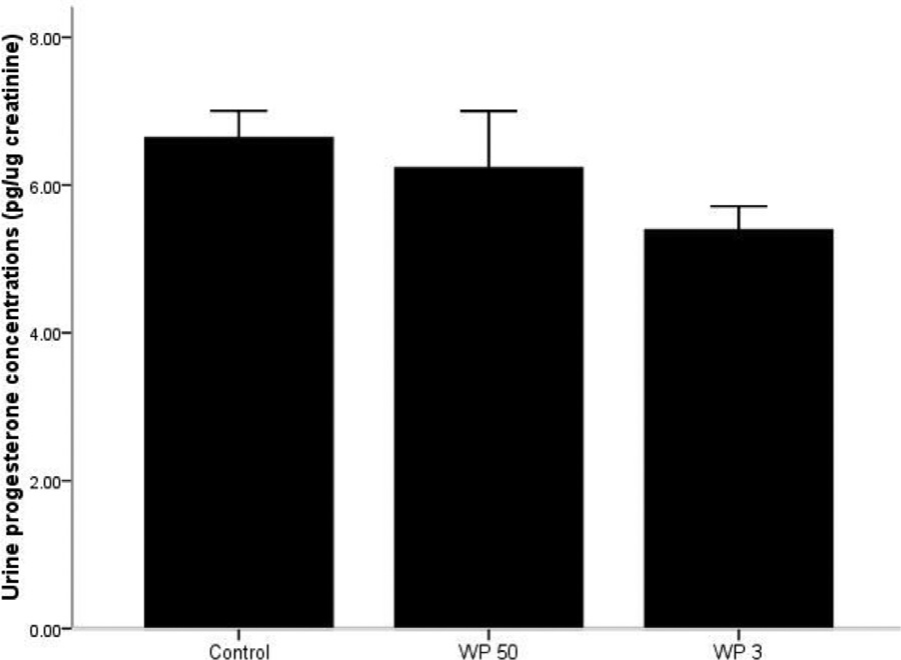
Urinary progesterone metabolites concentration in control (frozen urine) and 0th day Whatman 50 and 3 filter papers. Bars represent mean and standard deviation.

**Fig. 6:**
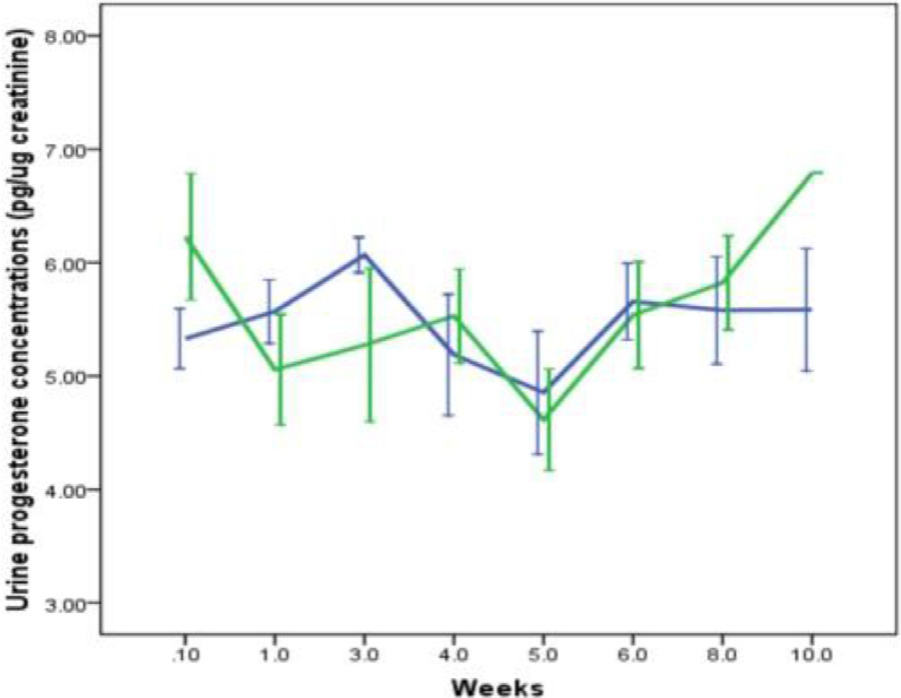
Urinary progesterone metabolites concentration from 0th week to 10th week stored on Whatman 50 (green line) and 3 filter papers (blue line). Bars represent mean and standard deviation

Similar to testosterone, there was significant variation in urine progesterone concentrations between control and two types of papers (K-W *X^2^*= 10.4; *p* = 0.007; Fig 5) following same day extraction. No significant difference was observed between control and WP 50 (M-W U test *U* = 6, *p* = 0.12) but a significant difference between the control and WP 3 (MW U = 1; *p* = 0.01) was observed. About 81.29% and 96.50% of urine progesterone (compared to control) were recovered from WP3 and WP 50 papers, respectively, following extractions on the same day. Interestingly, no significant difference was observed between two papers in the progesterone concentrations during the preservation period of 10 weeks (M- W Test U = 569; *p* = 0.021; Fig. 6). The intra-week co-efficient variations ranged from 1.1 to 11.4 while the inter week variation was 14.1 for WP 50. In the case of WP 3 the intra-week co-efficient ranged from 2.3 to 12.7 while the inter week variation was 6.6)

The present study reports a field method for short-term storage and preservation of anuran urine samples using Whatman filter papers grade 3 and 50. Overall, the Whatman filter paper grade 50 had a higher hormone recovery rate, more consistent and stable for short storage and preservation of anuran urine samples compared to grade 3 paper. The reason for better retention and the higher recovery rate in the WP 50 might be due to the high-quality cotton linters material that gives more wet-strength, high particle retention (2.7 um) and recrystallized of fine precipitates. However, WP3 was used successfully to store biological materials (proteins) for subsequent MALDI-TOF MS analysis [19]. Further, [20 showed better stability of thyroid hormones from blood spots imbibed on the filter paper when papers were stored at room temperature [20]. They also found that retention and recovery rate varied among the hormones following storage [20]. The WP 3 paper has also been used for storing blood samples for measurement of triglycerides in human [21]. As reported previously in this study also showed that the storage and retention of hormones in the paper varied among the hormones and paper qualities. No reports on WP 50 are available to compare with the present findings. Overall, the WP50 showed higher retention and recovery rate in both hormones might be due to the quality and retention characteristics of the paper [22]. Showed that storage of filter paper in room temperature did not affect hormone concentration up to one year, but lost hormone concentrations up to 50% but not on the quality of hormones following 5 years of storage. Further, they reported that creatinine concentrations in the stored papers were inconsistent and varied widely [22]. A lot of variations were also observed in the hormone concentrations because of different eluting solvents used. Knott 2005 demonstrated the use of absolute methanol as a potential solvent for eluting the steroid hormones from the filter paper and reported 86.4% recovery of estrone sulfate. Also, Knott 2005 found that estrone conjugates and creatinine extracted from dried paper significantly correlated with frozen control urine samples of orangutans following 2 years of storage [23].

In this study, Whatman 50 showed higher recovery of urinary progesterone (96.39%) and testosterone (85.06%) compared to control samples, resulting in a better paper for short-term preservation of urine samples at room temperature. Overall, the efficacy of urine storage and preservation, creatinine effective recovery, is crucial and depends on the property of paper, especially the particle retention pore size (crystalline retention), hardness and thickness of filter paper. Since creatinine is an indicator of the quality of urine and used as a concentration index, the paper must be chosen based on high retention characteristics. Collection of urine from free-ranging anurans using non-invasive method provides a crucial and effective way to monitor the reproductive physiology in the wild anurans. The results of the current study are a practical method to store and preserve the urine samples in field conditions. The advantage of this method is less expensive and no requirement for transportation of samples on dry ice or liquid nitrogen. This method allows the researchers to collect samples in remote areas for a long-term understanding of reproductive and stress physiology of a wide range of wild animals. However, a detailed study is required for long term storage of samples and their effect on quality and quantity hormones.

## Conclusion

The present study shows that desiccation of urine onto filter paper could be used as storage and preservation of anuran urine samples for reproductive hormone analysis up to 10 weeks. To our knowledge, first time anuran urine samples were stored onto Whatman filter papers (grade 50 and 3) for short term storage without refrigeration. High pressure liquid chromatography and enzyme immunoassays were validated and standardized to determine the efficacy of storage conditions by identifying and measuring the reproductive hormones (Progesterone and Testosterone) in urine samples. Whatman 50 grade paper found to be an effective, reliable and consistent filter paper for storage and preservation of anuran urine samples as compared to Whatman grade 3 filter paper. The advantage of this novel method is cost effective, simple storage method and could be used where limited logistics are available especially in the absence of refrigeration. Further, our results show that anuran urine could be stored at ambient temperature for a short-term period without significant loss of reproductive hormones in field conditions.
